# Mixed Scrub Typhus Genotype, Shandong, China, 2011

**DOI:** 10.3201/eid2003.121349

**Published:** 2014-03

**Authors:** Meng Zhang, Zhong-Tang Zhao, Xian-Jun Wang, Zhong Li, Lei Ding, Shu-Jun Ding, Li-Ping Yang

**Affiliations:** Shandong University, Jinan, China (M. Zhang, Z.-T. Zhao, L. Ding, L.P. Yang);; Shandong Center for Disease Control and Prevention, Jinan (X.-J. Wang, Z. Li, S.-J. Ding)

**Keywords:** scrub typhus, *Orientia tsutsugamushi*, coinfection, genotype, *Apodemus agrarius*, striped field mouse, HXS, sdu-2, bacteria, typhoid, scrub typhus, zoonoses, mite, vector, *Leptotrombidium arenicola*

**To the Editor:** Scrub typhus, which is caused by the bacterium *Orientia tsutsugamushi*, had been considered a disease of the tropical zone in China until the first outbreak in the temperate zone of Shandong Province in 1986 (*1*). The Sdu-2 genotype had not been reported for human patients in China since its detection in the striped field mouse (*Apodemus agrarius*) during 2007 (*2*). We report a case in a person in Shangdong Province who was co-infected with the HSX and Sdu-2 genotypes of *O. tsutsugamushi.*

A female farmer, 55 years of age, was admitted to Mengyin County Hospital on September 29, 2011, with a 5-day history of fever (highest temperature 39°C), headache, back pain, chills, anorexia, and nausea. Her back pain was exacerbated as her fever increased. A black eschar (10 mm × 20 mm) was observed above the left breast, and 2 enlarged lymph nodes (20 mm × 30 mm) that were tender to the touch were found behind the right ear. The patient’s Glasgow Coma Scale score was 15. No comorbidities were reported. Laboratory tests showed slight leukopenia, low potassium level, decreased albumin/globulin ratio, elevated lactic dehydrogenase level, and elevated C-reactive protein level. Results of urine dry chemical analysis were positive for occult blood and leukocytes. The Weil-Felix reaction to the *Proteus* OXK antigen was negative. Mild abnormalities were observed in results of an electroencephalogram and an electroencephalogram topographic map. The patient was suspected to have scrub typhus complicated by encephalitis and urinary tract infection. Doxycycline (200 mg once daily) and symptomatic treatment were administered immediately. The patient’s symptoms had generally subsided on day 5 of hospitalization. Indirect immunofluorescence assay showed a 4-fold rise in IgG titer against *O. tsutsugamushi* between acute-phase (1:128) and convalescent-phase (1:512) serum samples collected 18 days apart.

Acute-phase whole blood was collected before administration of antimicrobial drugs. The eschar was collected after natural desquamation. DNA from the whole blood and eschar was isolated and screened for *O. tsutsugamushi* by using PCR primers E and B to target the 56-kDa type-specific antigen gene (*3*). Type-specific amplification of *O. tsutsugamushi* was performed (*4*,*5*). Normal values for fragment yield are 407, 230, 242, 220, 600, and 523 bp for Gilliam, Karp, Kato, Kuroki, Saitama, and Kawasaki genotypes, respectively.

Three products that had the same molecular weight as those from Kato, Saitama, and Kawasaki genotypes were generated from the blood sample by using type-specific amplification. However, sequence analysis of amplicons yielded by using type-specific primers and those yielded by using primers E and B demonstrated a co-infection in the blood with 2 genotypes of *O. tsutsugamushi*, which were designated as ZZF-KW (661 bp, GenBank accession no. JX644590) and ZZF-HSB (498 bp, GenBank accession no. JX644591). Co-infection with the 2 genotypes was also detected in the eschar. BLAST (http://blast.ncbi.nlm.nih.gov/Blast.cgi) analysis showed that ZZF-KW had 100% sequence similarity to the HSX genotype of *O. tsutsugamushi* (657 bp, GenBank accession no. JX202566) and that ZZF-HSB had 100% sequence similarity to Sdu-2 genotype (476 bp, GenBank accession no. EF543196). A nucleotide identity of 72% was shown between ZZF-KW and ZZF-HSB. Nucleotide identities of partial 56-kDa type-specific antigen genes among the 2 *O. tsutsugamushi* genotypes and the reference types are shown in the Table.

**Table:** Nucleotide sequence homologies of partial 56-kDa type-specific antigen gene sequences among *Orientia tsutsugamushi* genotypes and reference strains, China, 2011

Genotype	*O. tsutsugamushi* reference strains, % identity							
	HSX	Sdu-2	Gilliam	Karp	Kato	Kawasaki	TA686	HSB1
ZZF-KW	100	68.8	90.3	72.7	73.8	96.0	67.3	74.7
ZZF-HSB	68.8	100	68.1	72.1	66.6	67.7	77.8	74.3

Co-infection with HSX and Sdu-2 genotypes of *O. tsutsugamushi* was detected in a single case. Mixed genotype infections could not be detected by using 1 amplicon, which could amplify only a predominant genotype. Type-specific amplification is a convenient method for the detection of mixed genotype infection, although this method has limitations for identification of novel genotypes. The Sdu-2 genotype was not differentiated from Kato, Saitama, and Kawasaki genotypes by using type-specific primers, but differentiation was achieved by gene sequencing. Considering the genotypic diversity (*6*) and mixed genotype infection of the agent, type-specific primers require redesign for type designation and subsequent sequencing is recommended for verification.

Coexistence of multiple genotypes *O. tsutsugamushi* was reported for 25% of scrub typhus patients in a study population in Thailand (*7*). Mixed genotype infections of a single pathogen species in the ecosystem are common; these mixed genotypes provide possibilities for recombination events and prompts the coevolution of host–parasite interactions (*8*). Disease severity and epidemiologic characteristics could be influenced by ecologic interactions between genetically diverse strains (*8*). In this report, we describe a single case; comparison of disease severity of scrub typhus caused by mixed and single genotype infections should be studied further.

Simultaneous infection with multiple antigenic strains of *O. tsutsugamushi* was detected in an individual mite, *Leptotrombidium arenicola* (*9*), a probable vector of scrub typhus. Infection with multiple *O. tsutsugamushi* strains may be caused by being bitten by multiple mites or by multiple genotypes coexisting within individual mites (*7*). We ascribed the co-infection to the second cause because the 2 genotypes were simultaneously detected from an eschar sample associated with the bite of 1 mite examined in this study. There may be diverse genotypic co-infection patterns of *O. tsutsugamushi*. Mechanisms of in-host interactions between genetically diverse strains of *O. tsutsugamushi* and the initiated host response require the establishment of animal models for further research.

## References

[1] Yang YF, Wang JL, Yao YC. Investigation of the first scrub typhus epidemic in Shandong Province [in Chinese]. Zhonghua Liu Xing Bing Xue Za Zhi. 1987;8:280.

[2] Yang LP, Zhao ZT, Li Z, Wang XJ, Liu YX, Bi P. Comparative analysis of nucleotide sequences of *Orientia tsutsugamushi* in different epidemic areas of scrub typhus in Shandong, China. Am J Trop Med Hyg. 2008;78:968–72 . 18541778

[3] Enatsu T, Urakami H, Tamura A. Phylogenetic analysis of *Orientia tsutsugamushi* strains based on the sequence homologies of 56-kDa type-specific antigen genes. FEMS Microbiol Lett. 1999;180:163–9 . . 10.1111/j.1574-6968.1999.tb08791.x 10556707

[4] Furuya Y, Yoshida Y, Katayama T, Yamamoto S, Kawamura A Jr. Serotype-specific amplification of *Rickettsia tsutsugamushi* DNA by nested polymerase chain reaction. J Clin Microbiol. 1993;31:1637–40 . 8315007 10.1128/jcm.31.6.1637-1640.1993 PMC265595

[5] Tamura A, Yamamoto N, Koyama S, Makisaka Y, Takahashi M, Urabe K, Epidemiological survey of *Orientia tsutsugamushi* distribution in field rodents in Saitama Prefecture, Japan, and discovery of a new type. Microbiol Immunol. 2001;45:439–46 . 10.1111/j.1348-0421.2001.tb02643.x 11497219

[6] Kelly DJ, Fuerst PA, Ching WM, Richards AL. Scrub typhus: the geographic distribution of phenotypic and genotypic variants of *Orientia tsutsugamushi.* Clin Infect Dis. 2009;48(s3):Suppl 3:S203–30. 10.1086/596576 19220144

[7] Sonthayanon P, Peacock SJ, Chierakul W, Wuthiekanun V, Blacksell SD, Holden MT, High rates of homologous recombination in the mite endosymbiont and opportunistic human pathogen *Orientia tsutsugamushi.* PLoS Negl Trop Dis. 2010;4:e752 . . 10.1371/journal.pntd.0000752 20651929 PMC2907413

[8] Read AF, Taylor LH. The ecology of genetically diverse infections. Science. 2001;292:1099–102 . . 10.1126/science.1059410 11352063

[9] Shirai A, Huxsoll DL, Dohany AL, Montrey RD, Werner RM, Gan E. Characterization of *Rickettsia tsutsugamushi* strains in two species of naturally infected, laboratory-reared chiggers. Am J Trop Med Hyg. 1982;31:395–402 . 6176132 10.4269/ajtmh.1982.31.395

